# Invasive Fungal Laryngotracheitis Causing Laryngotracheal Separation

**DOI:** 10.7759/cureus.71558

**Published:** 2024-10-15

**Authors:** Nazirah Baharudin, Vanitha Palanisamy, Mawaddah Azman, Kishanti Balachandran, Farhana Arif

**Affiliations:** 1 Department of Otolaryngology - Head and Neck Surgery, Hospital Kuala Lumpur, Kuala Lumpur, MYS; 2 Department of Otolaryngology - Head and Neck Surgery, Faculty of Medicine, Universiti Kebangsaan Malaysia, Kuala Lumpur, MYS; 3 Department of Radiology, Hospital Shah Alam, Shah Alam, MYS; 4 Department of Otorhinolaryngology, Hospital Shah Alam, Shah Alam, MYS

**Keywords:** antifungal drugs, candida guillermondii, fungal laryngotracheitis, laryngotracheal separation, opportunistic infection

## Abstract

Fungal laryngotracheitis (FLT) is rare, and the diagnosis can be challenging, as its presentation lacks specificity and may resemble other conditions such as granulomatous disease, gastroesophageal reflux, or malignancy. FLT can be very invasive, causing complete laryngotracheal separation, leading to a non-functioning larynx. We report a 39-year-old Indian woman with diabetes who presented to the emergency department with a sore throat, hoarseness, dysphagia, and stridor for two days. Initially treated for diabetic ketoacidosis due to acute tonsillopharyngitis, she required intubation for airway obstruction and severe metabolic acidosis. Fourteen days post-intubation, an airway assessment revealed bilateral vocal fold edema and pus in the subglottic and cervical trachea. CT imaging showed circumferential fluid around the trachea and a distorted larynx. Examination under anesthesia and neck exploration revealed pus around the thyroid gland and trachea, leading to a tracheostomy and sample collection. Histopathology indicated a fungal infection, confirmed as *Candida guillermondii* with *Escherichia coli*. The patient was treated with oral fluconazole and intravenous cefuroxime for four weeks. Despite treatment, a repeat CT indicated a non-functioning larynx, prompting a proposal for a total laryngectomy. After a multidisciplinary discussion, it was decided to continue antifungal therapy due to the patient's clinical improvement. At follow-up a month later, she was stable, tolerating oral intake with a double-lumen tracheostomy tube. This case underscores the importance of a high index of suspicion for FLT and the need for patient-specific decisions regarding total laryngectomy in a non-functioning larynx.

## Introduction

Fungal laryngotracheitis (FLT) is a rare clinical phenomenon [1-6]. The prevalence of this opportunistic infection affecting the larynx has risen due to the widespread use of steroid therapy and antibiotics, alongside an increasing population of immunocompromised patients [5,6]. Diagnosis of FLT poses a challenge due to its nonspecific presentation, often resembling other conditions such as granulomatous disease, gastroesophageal reflux, or malignancy. A delayed diagnosis of FLT often leads to significant morbidity for the patient. Management of this rare disease is often individualized and requires multidisciplinary team involvement. In this case report, we aim to emphasize the presentations, diagnosis, and treatment options for FLT presenting with a non-functioning larynx.

## Case presentation

**Figure 1:**
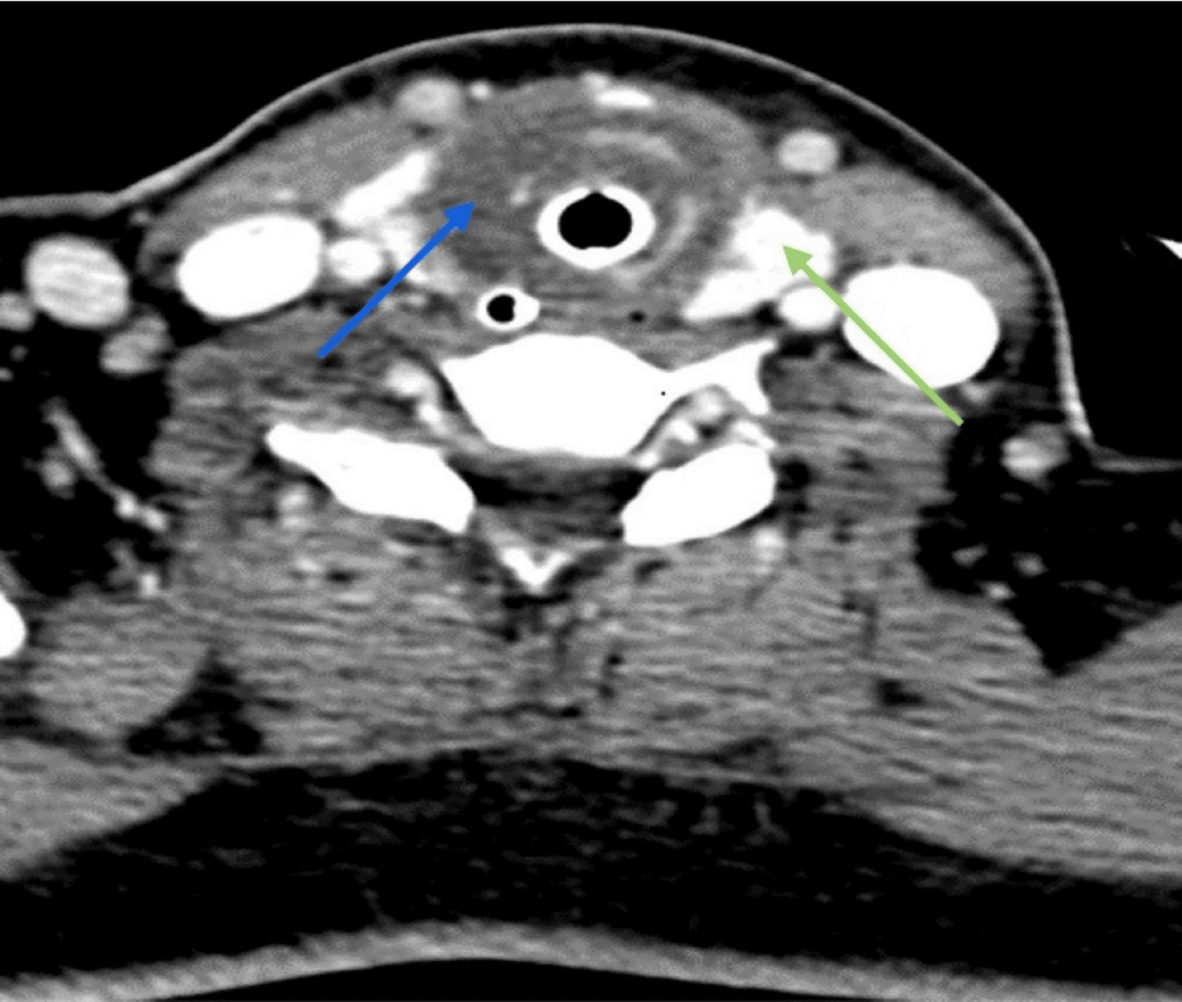
CT axial view shows circumferential thin fluid seen surrounding the trachea (blue arrow), adjacent to the thyroid gland (green arrow).

**Figure 2:**
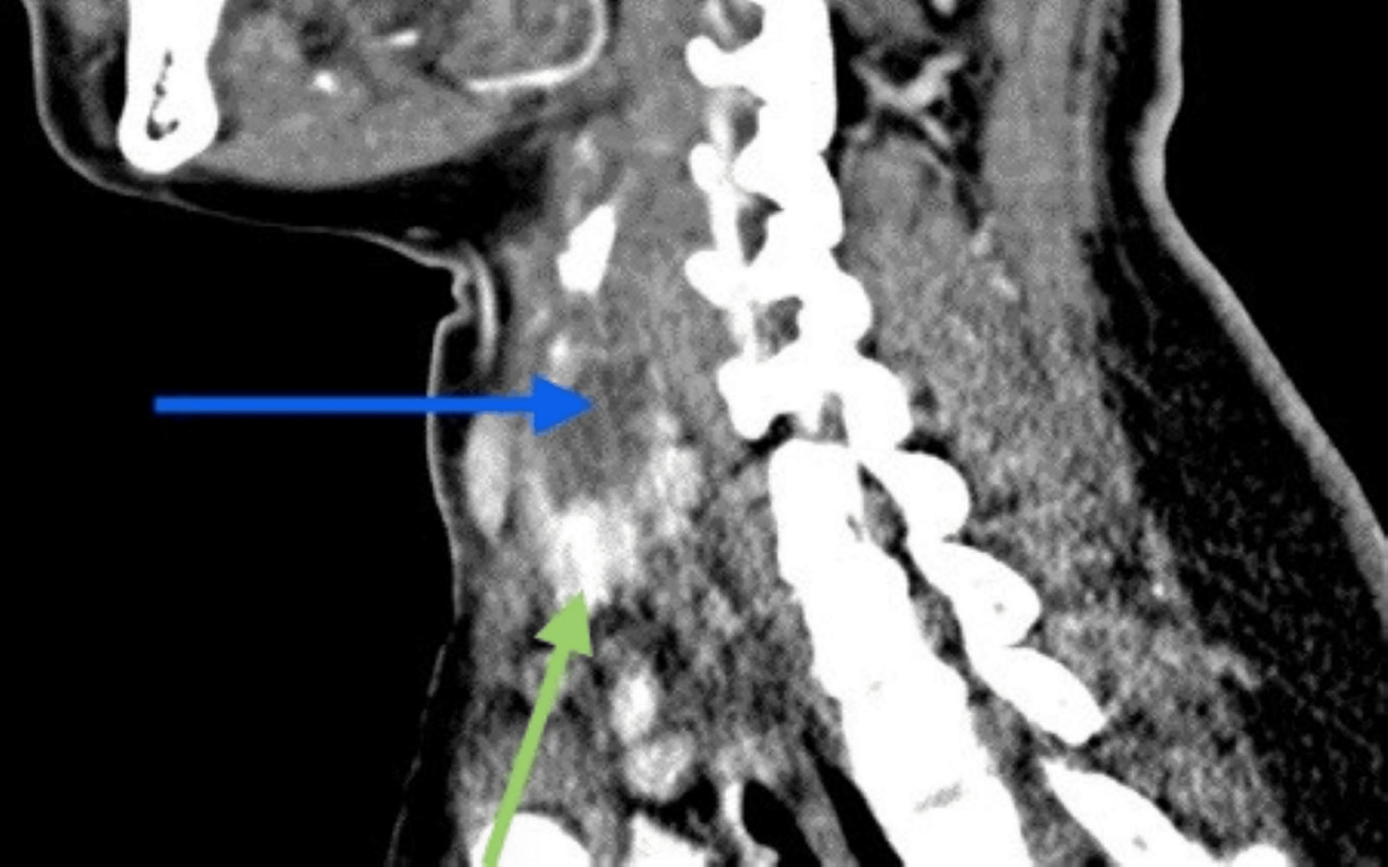
CT sagittal view shows circumferential thin fluid seen surrounding the trachea (blue arrow), adjacent to the thyroid gland (green arrow).

A 39-year-old Indian lady with underlying diabetes mellitus and dyslipidemia presented with a two-day history of sore throat, stridor, hoarseness, and dysphagia. It was preceded by a chesty cough for one week. Upon presenting at the emergency department, her capillary blood glucose level was high, and arterial blood gas showed metabolic acidosis. The flexible scope showed bilateral vocal cord edema and injected pharynx. The patient was treated for diabetic ketoacidosis secondary to acute tonsillopharyngitis. She was intubated for impending airway collapse and severe metabolic acidosis. She required a high dose of antibiotics to treat the sepsis. She was not able to wean off the ventilation, and her infective markers were not improving. A CT scan of the neck showed circumferential thin fluid surrounding the proximal trachea at the C6 level adjacent to the thyroid gland (Figures 1, 2) with no clear plane between the fluid and the adjacent thyroid parenchyma.

**Figure 3:**
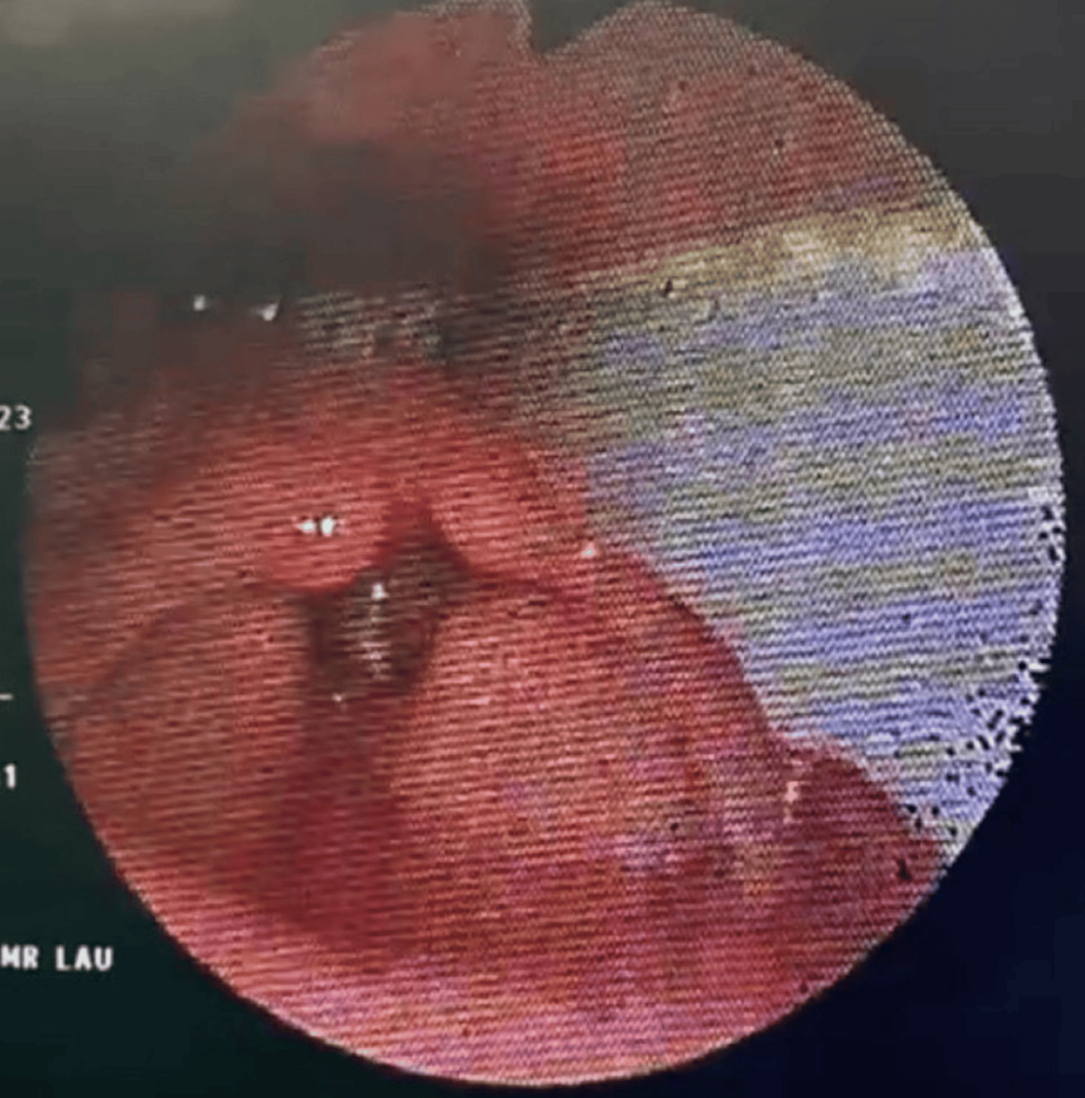
Bilateral vocal fold edema seen during EUA. EUA: examination under anesthesia

**Figure 4:**
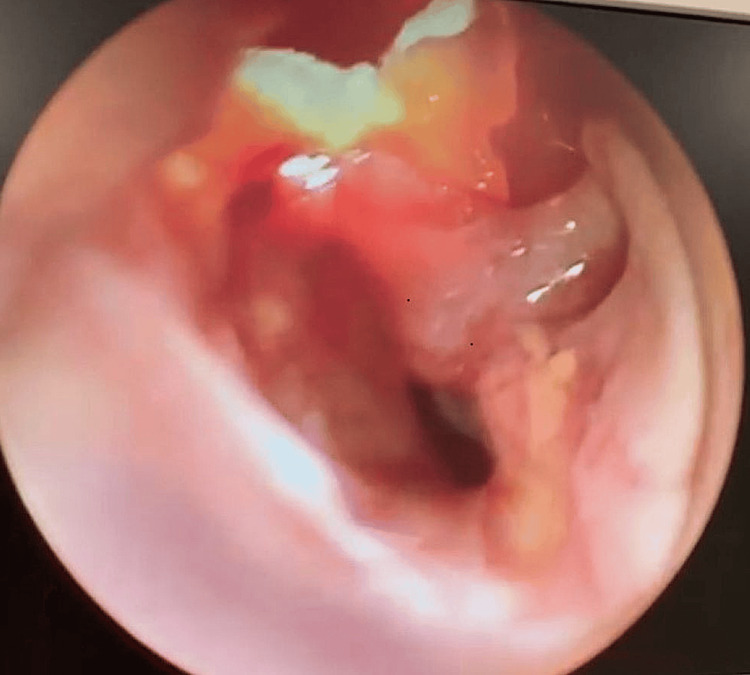
Thick slough and pus covering the subglottic region and cervical trachea airway.

An EUA (examination under anesthesia) revealed bilateral vocal fold edema and the presence of pus in the subglottic region, as shown in Figures 3, 4.

Transcervical neck exploration was performed, revealing the presence of pus surrounding the thyroid gland and the trachea. A malacic segment of the anterior tracheal wall was present due to the infection; a tracheostomy was performed, and samples sent for examination revealed *Candida guillermondii* with a superimposed *Escheria Coli* infection. The patient was administered oral fluconazole 400 mg OD and intravenous cefuroxime 750 mg TDS for a total duration of four weeks.

**Figure 5:**
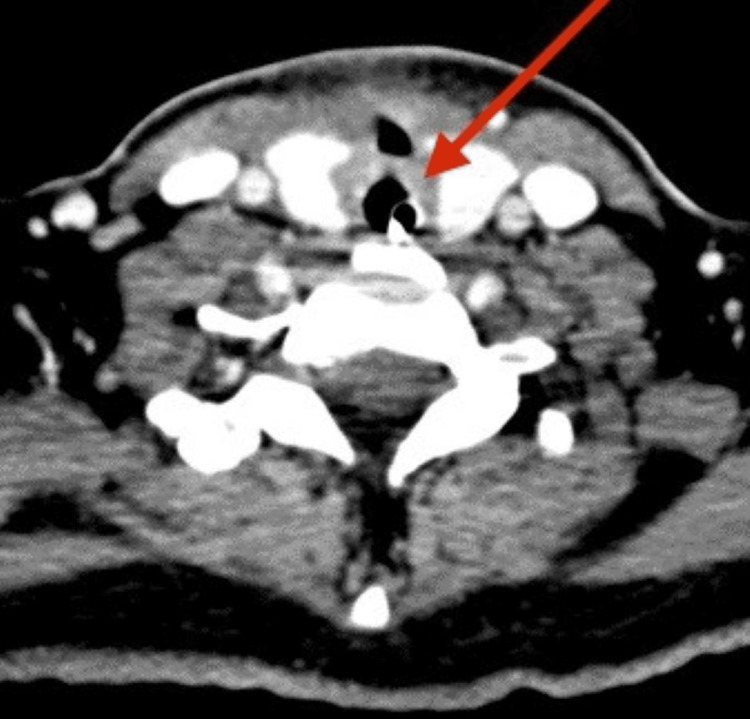
CT axial view shows the resolution of previously seen fluid along the proximal trachea (red arrow).

**Figure 6:**
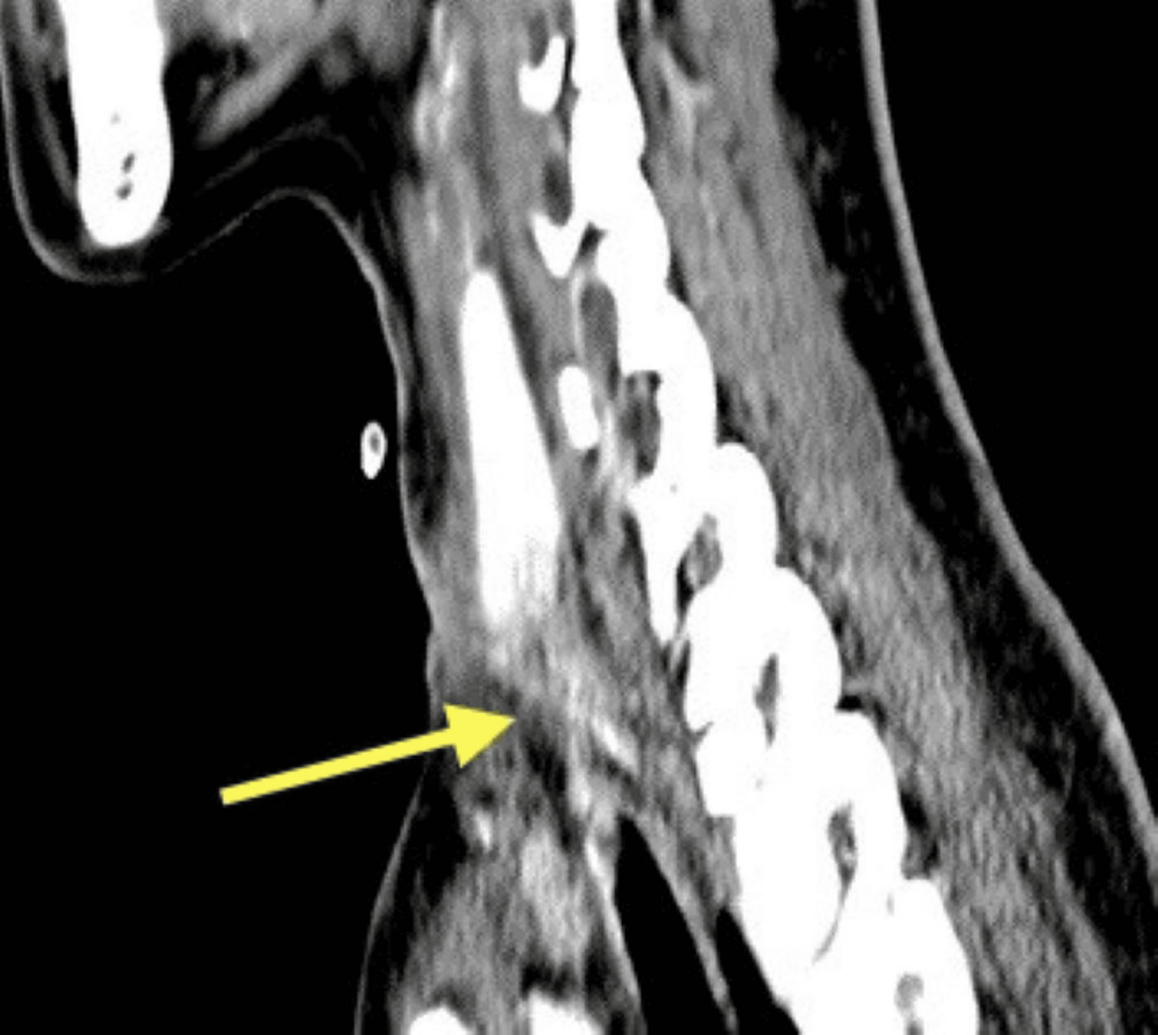
CT sagittal view: the laryngotracheal area was obliterated due to soft tissue edema, the thyroid cartilage was eroded, and the cricoid cartilage was absent (yellow arrow).

A repeat CT a month later showed worsening airway edema with erosion of the thyroid and cricoid cartilage with resolution of the previously seen fluid along the proximal trachea (Figure 5). However, the laryngotracheal area was obliterated due to soft tissue edema. The thyroid cartilage was eroded, and the cricoid cartilage was absent (Figure 6). Upper tracheal rings were also distorted, and the upper tracheal ring just above the tracheostomy was almost completely obliterated. The trachea at the tracheostomy until the C7/T1 level was distorted and slightly narrowed, while the remaining of the lower trachea, carina, and main bronchi remained patent.

**Figure 7:**
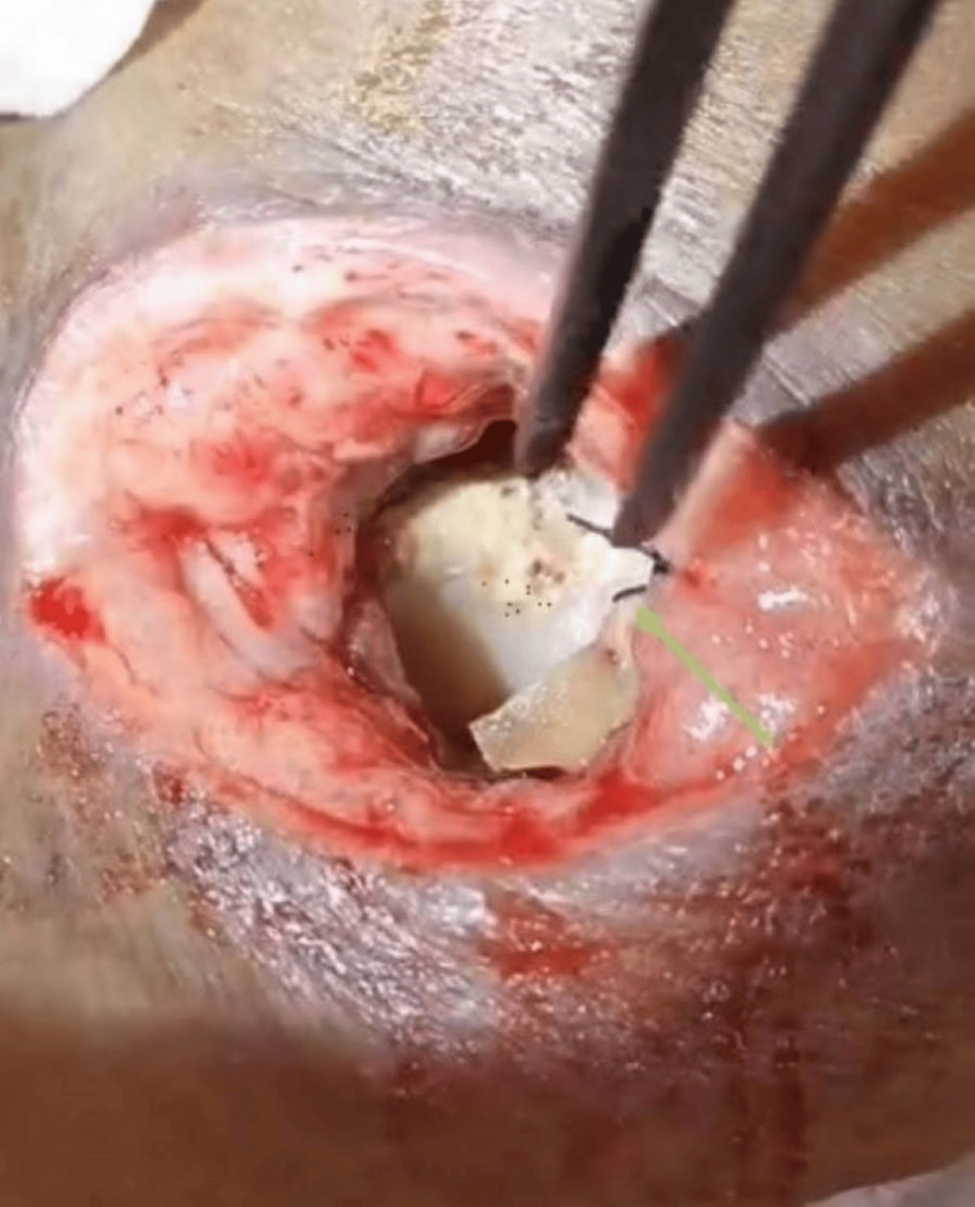
As seen from the tracheostoma, the remaining posterior cricoid was sutured to the strap muscle (green arrow).

**Figure 8:**
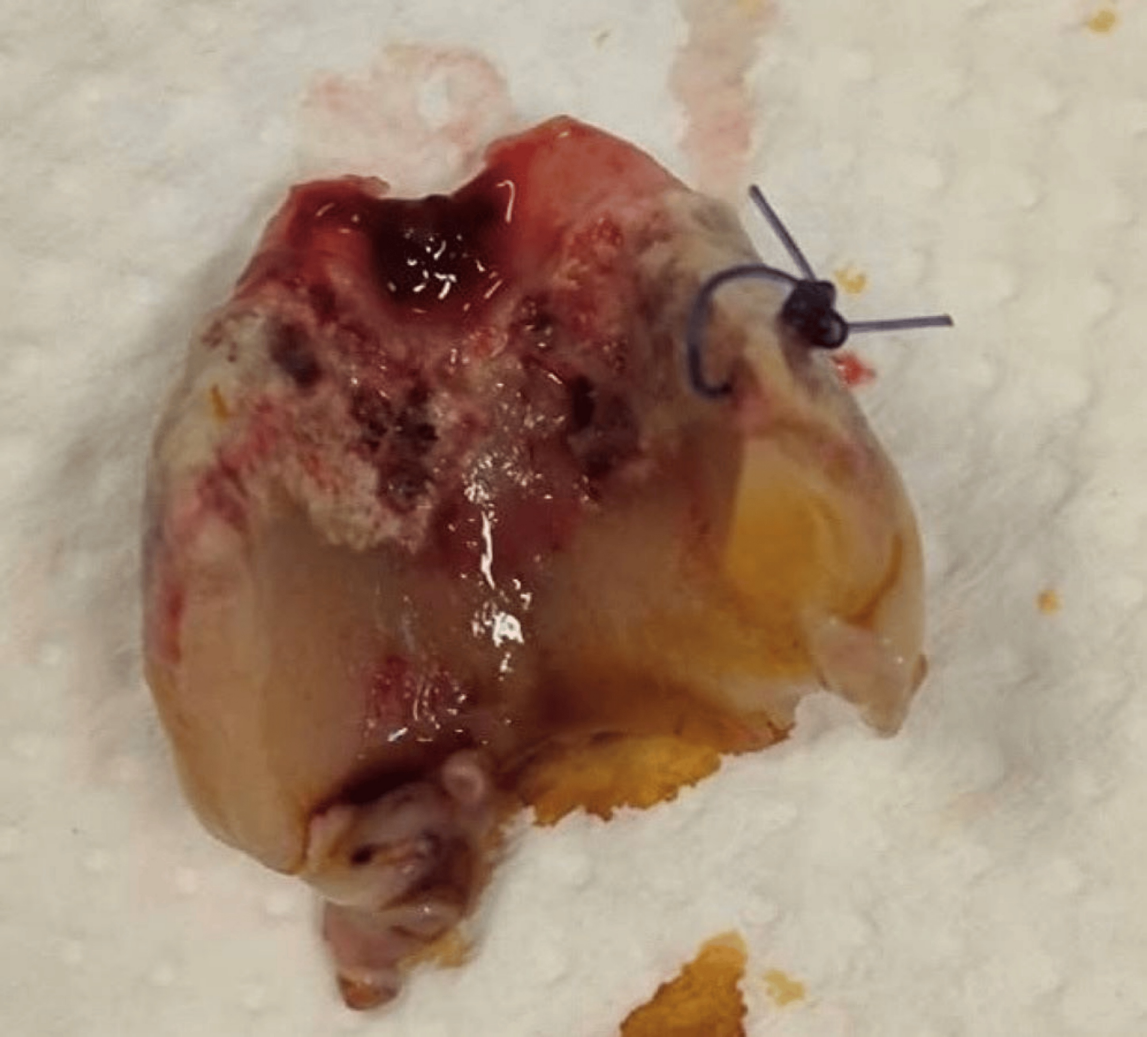
Removal of the sloughy and necrotic part of the cricoid cartilage, which was detached from the thyroid cartilage.

Direct laryngoscopy and esophagoscopy were also performed four weeks posttreatment. Intraoperative findings showed worsening distortion of the larynx. The vocal cord was edematous at the median position, with the glottic region showing sloughing extending to the suprastoma. The cricoid cartilage was detached from the thyroid cartilage. There is a break at the anterior malacic cricoid cartilage, and the posterior cricoid ring is partially eroded. The first and second tracheal rings are malacic and in a distorted shape. The mucosa below the stoma until the carina appears inflamed, with minimal slough. The remaining cricoid cartilage was sutured to the strap muscle (Figure 7). The sloughy and necrotic part of the cricoid cartilage was removed (Figure 8). The esophagoscopy finding showed the anterior esophagus wall to be erythematous at a distance of 16 cm from the incisor.

Despite the worsening of the larynx findings, the patient remains stable on the tracheostomy tube with no respiratory distress and no fever. The inflammatory markers showed a decreasing trend. The patient was put on Ryle's tube feeding to optimize her nutrition and avoid aspiration. On swallowing, assessment showed no sign of aspiration, and the patient was able to slowly take orally. The dilemma at hand was whether to eliminate the source of infection with total laryngectomy, risking poor wound healing, or pursue conservative management.

Following a multidisciplinary discussion, the patient and physicians concurred to pursue conservative treatment with long-term antifungal therapy, alongside monitoring of infective indicators and imaging. A subsequent scan showed no structural changes or inflammatory processes. C-reactive protein (CRP) levels down to single digits indicated resolution of infection. The patient was taken off the antifungals, and she is able to tolerate a normal diet without difficulties. For long-term airway management, we plan to replace her tracheostomy tube with a double-lumen tracheostomy tube to maintain airway patency.

This case study aims to examine the clinical findings of FLT, the investigative procedures, and the surgical and medical treatment options for a case of laryngotracheal discontinuation.

## Discussion

FLT is more common in middle-aged males between 41 and 60 years old [7]. Among the fungal species identified that involved the upper airway are candida, blastomycoses, histoplasmoses, coccidioidomycosis, paracoccidioidomycosis, and aspergillus [7]. Candida, which is part of the normal flora of the mucosa in our oral cavity and pharynx, is the most common opportunistic organism that colonizes the respiratory tract and is also one of the causative agents of FLT [8]. In our case report, *Candida guillermondii* was detected from histopathology and can be invasive, particularly in immunosuppressed patients. Treating *Candida guillermondii* is a challenge as it has low susceptibility to azoles and echinocandins [8]. Fungal colonization impairs the immune response, hence permitting secondary bacterial infection. Among the most prevalent bacterial pathogens are *Streptococcus pneumoniae, Haemophilus influenza*, and *Staphylococcus aureus* [9]. In our case, the FLT infection was superimposed with *Escherichia coli*.

The presentation of FLT is similar to other diseases, such as granulomatous disease, gastroesophageal reflux, or malignancy, resulting in delayed diagnosis and not being treated promptly. In severe cases, they may present with hoarseness, dysphagia, odynophagia, sore throat, or respiratory distress [4]. Among all these, hoarseness is the main complaint presented [5,10]. The duration of symptoms can range from weeks to months. As in our case, the patient presented rather acutely with respiratory distress, which required intubation. The diagnosis of FLT was not made until two weeks after failed extubation and until the patient had undergone an airway assessment by an otorhinolaryngologist. Hence, a high index of suspicion is needed to diagnose FLT, especially in immunocompromised patients.

Our body's primary defense mechanism against fungus is phagocytosis by macrophages and granulocytes [7]. Hence, factors that alter the immune response, such as diabetes mellitus, immunosuppressive medications such as chemotherapy, systemic corticosteroids, nutritional deficiency, AIDS, and chronic lymphocytic leukemia, predispose the person to fungal infections [9]. Other predisposing factors include altered mucosal barriers from various causes, including radiotherapy, inhaled corticosteroids, gastroesophageal reflux, any history of trauma such as previous intubation, and smoking [9]. In our case, the only predisposing factor present is her underlying diabetes mellitus. The patient was diagnosed with diabetes mellitus in the past two years but, unfortunately, was not compliant with her medications. The most common site involved in fungal larynx infection is the true vocal cord [7,10]. Furthermore, there is a clear predominance of glottic infection when compared to other laryngeal subsites, which is likely due to the fact that this is the narrowest aspect of the adult airway and is the site of maximum vibration of the vocal folds, hence leading to mucosal trauma and microsulci formation, thus most susceptible to mycotic infection [3].

Flexible nasopharyngolaryngoscopy findings can suggest a diagnosis of fungal laryngitis. However, fungal lesions may manifest as irregular whitish patches, posing a risk of misdiagnosis with other laryngeal leukoplakia lesions such as hyperkeratosis, verrucous growths, or squamous cell carcinoma [7]. In cases of FLT, the vocal cords can exhibit erythematous, edematous, ulcerated conditions and, occasionally, the formation of pseudomembranes [5]. Fungal infiltration into the basement membrane can induce stiffness in the vocal cords, resembling early signs of laryngeal cancer [7]. In our specific case, FLT displayed an invasive nature, resulting in severe erosion and destruction of the thyroid and cricoid cartilage, as well as the first and second tracheal rings. The vocal cords exhibited pronounced edema, and the presence of slough tissue raised suspicions of a fungal infection.

There are a few ways FLT can be diagnosed. Non-invasive investigations include flexible fiberoptic nasopharygolaryngoscopy, as mentioned previously; however, due to its non-specificity, it is not a definite diagnosis tool that can be used. The definitive diagnosis of fungal laryngitis is the presence of fungal spores, hyphae, or pseudohyphae in culture or tissue biopsy [3-6,9]. Besides diagnosing FLT, a biopsy can also help rule out malignancy or other diseases of the larynx. Some of the staining methods used in identifying fungi include Gomori methenamine silver (GMS), Gram-staining, and periodic acid-Schiff staining (PAS) [11]. As in our case, the fungal elements were highlighted by PAS and GMS staining. Other advancements in molecular testing, such as fungal polymerase chain reaction (PCR), have been used to expedite the identification of fungal infections [1].

The cornerstone of FLT management is antifungal therapy. However, surgical debridement is indicated in selected invasive cases, with treatment outcomes frequently focusing on the eradication of disease rather than preserving voice outcomes. Optimization of the predisposing factors is one of the important key management strategies in FLT; for instance, in our patient, the optimization of her glucose level is important in boosting immunity and wound healing. Most authors treated their patients with antifungal alone with a minimum duration of one month to 12 months without requiring extensive surgery [2-7]. To our knowledge, there are no local guidelines for the use of antifungals and their duration for FLT. Morse et al. [3] reported isolated primary laryngeal cryptococcus, and they treated their patient with antifungal for 12 months and attained full resolution of symptoms by six months of treatment but continued the treatment until 12 months after normalization of serum cryptococcal antigen. Subglottic involvement may represent a higher infectious burden, hence requiring longer treatment of antifungal [3].

According to the Infectious Diseases Society of America (IDSA), four types of antifungals can be used, including polyenes, amphotericin B (AmB) deoxycholate (AmB-d) or lipid formulation amphotericin B (L-AmB), AmB lipid complex (ABLC), and AmB colloidal dispersion (ABCD), the triazoles (fluconazole, itraconazole, voriconazole, and posaconazole), the echinocandins (caspofungin, anidulafungin, and micafungin), and flucytosine. For invasive candidiasis, the intravenous dosage for AmB-d is 0.5-0.7 mg/kg daily, but dosages as high as 1 mg/kg daily should be considered [10]. The most common side effects of AmB are nephrotoxicity. However, the availability of lipid formulation AmB offers enhanced renal protection but is more costly. Hence, it should only be considered in patients with renal failure.

It is imperative that we collaborate closely with the infectious disease team to manage patient cases individually. According to IDSA, triazoles such as fluconazole, itraconazole, voriconazole, and posaconazole demonstrate similar activity against most Candida species. Fluconazole also exhibits similar efficacy to that of AmB-d for the treatment of candidemia [10]. Fluconazole is also considered the standard therapy for oropharyngeal, esophageal, and vaginal candidiasis [10]. The recommended dose for patients with invasive candidiasis is a loading dose of 800 mg (12 mg/kg), followed by a daily dose of 400 mg (6 mg/kg), whereby a lower dosage is required in patients with creatinine clearance <50 mL/minute. In our case, 400 mg of oral fluconazole was administered daily for a duration of two months. We planned to continue anti-fungal treatment and conduct a repeat CT scan in a month. In our case report, some symptoms were resolved after a month of oral antifungal, as evidenced by a reduction in inflammatory markers. However, there was a progression of destruction in her laryngeal framework, resulting in her inability to vocalize.

Although surgical debridement is acknowledged as a treatment for apparent necrosis, the most effective extent and timing of debridement necessary to achieve optimal results have yet to be established [4]. In our case, the patient not only developed invasive FLT, resulting in necrosis of the larynx, but it is so extensive that it caused complete laryngotracheal separation. The cricoid cartilage is detached from the thyroid cartilage. The necrotic thyroid cartilage was eroded, while the cricoid cartilage partially dissolved and was absent one month after the onset of symptoms (Figure 8).

A similar case was reported by Swiss et al. [12], where they repeatedly performed a surgical debridement on their patient via direct laryngoscopy and through a 5 cm horizontal transcervical incision over the cricothyroid membrane with concomitantly giving intravenous liposomal AmB. The aim of debridement is to remove the necrotic debris; however, despite undergoing debridements, the disease is so severe that the larynx eventually becomes non-viable, and total laryngectomy was considered. However, it was not performed due to patient comorbidities [12]. The patient acquired a pharyngotracheal fistula due to stress from oral feeding; however, she remained stable and was able to whisper during her subsequent follow-up.

In another case report by Lapointe et al., a patient with underlying leukemia presented with a fungal abscess of the larynx, prompting a total laryngectomy and right hemithyroidectomy due to inadequately controlled symptoms despite high doses of steroids, broad-spectrum antibiotics, and antifungal treatment. They continued triple antifungal for six months, and the patient was disease-free after 12 months of follow-up.

In cases where the larynx has become non-functional, a total laryngectomy can be considered to remove the source of infection. However, this decision requires a multidisciplinary team involvement as major surgical procedures have significant consequences for the patient's quality of life. In our case, given the patient's poor wound healing, the decision is made to continue with oral antifungal treatment, monitor her inflammatory markers, and repeat the CT scan. Fortunately, the patient responded well to her treatment and showed promising disease control on her subsequent follow-up.

## Conclusions

In conclusion, invasive FLT represents a rare yet serious condition typically observed in immunocompromised patients. The management of such cases necessitates a multifaceted approach involving antifungal and antibacterial therapies, especially in the context of superimposed bacterial infections. Surgical intervention, such as debridement, plays a crucial role in controlling the infection and improving patient outcomes. Interestingly, this case report highlights that total laryngectomy may not always be obligatory, particularly in scenarios involving a non-functioning larynx or laryngotracheal separation. Such findings underscore the importance of individualized treatment strategies tailored to the unique circumstances of each patient, aiming not only to eradicate the infection but also to preserve functional integrity whenever feasible.
